# Assessing the scales in numerical weather and climate predictions: will exascale be the rescue?

**DOI:** 10.1098/rsta.2018.0148

**Published:** 2019-02-18

**Authors:** Philipp Neumann, Peter Düben, Panagiotis Adamidis, Peter Bauer, Matthias Brück, Luis Kornblueh, Daniel Klocke, Bjorn Stevens, Nils Wedi, Joachim Biercamp

**Affiliations:** 1 Deutsches Klimarechenzentrum, Bundesstr. 45a, 20146 Hamburg, Germany; 2 European Centre for Medium-Range Weather Forecasts, Shinfield Park, Reading RG2 9AX, UK; 3 Max-Planck-Institut für Meteorologie, Bundesstr. 53, 20146 Hamburg, Germany; 4 Deutscher Wetterdienst, Frankfurter Str. 135, 63067 Offenbach, Germany

**Keywords:** global high-resolution modelling, ICON, IFS, scalability, exascale computing

## Abstract

We discuss scientific features and computational performance of kilometre-scale global weather and climate simulations, considering the Icosahedral Non-hydrostatic (ICON) model and the Integrated Forecast System (IFS). Scalability measurements and a performance modelling approach are used to derive performance estimates for these models on upcoming exascale supercomputers. This is complemented by preliminary analyses of the model data that illustrate the importance of high-resolution models to gain improvements in the accuracy of convective processes, a better understanding of physics dynamics interactions and poorly resolved or parametrized processes, such as gravity waves, convection and boundary layer.

This article is part of the theme issue ‘Multiscale modelling, simulation and computing: from the desktop to the exascale’.

## 1. Introduction

Weather predictions and climate projections are of vital importance for society and for the creation and preservation of prosperity. One important component of these predictions is numerical models that simulate the dynamics of several parts of the Earth System (including atmosphere, ocean, sea ice, land ice and vegetation), and their interactions. Owing to the number of relevant physical processes, their complexity and the high dimensionality of the Earth System, weather and climate models are supercomputing applications, consisting of millions of lines of code, and model resolution is limited by available computational power.

*Operational weather forecasts* run several times per day under very tight schedules that include the collection and processing of observational data, data assimilation to create initial conditions for forecasts, the integration of the forecast model, data post-processing and dissemination of forecast products to users. This complex schedule imposes timing requirements on forecast runs to complete within about 1 hour. This translates to execution rates of about 200–300 forecast days per wall clock day, which is close to one simulated year per day (SYPD), using several hundreds of compute nodes for deterministic runs and about a thousand nodes for ensembles, resembling tens of thousands of compute cores. For weather forecast models, different model configurations are used for different predictions. Predictions into the medium-range (e.g. forecast days 3–15) require global models to cover scale interactions and interactions between different physical processes all over the planet. Current forecasts run at about 10 km spatial resolution for single deterministic predictions and at about 20 km resolution for ensembles. For short forecasts of several days, global scale interactions are less important and forecasts with limited-area models are performed at about one order of magnitude higher spatial resolution. However, high-resolution limited-area models also rely on global predictions because they require boundary and initial conditions.

*Models for climate projections* are run in a different set-up as long integrations designed to explore factors influencing energy exchanges. Traditionally, fluid-dynamical processes, which dominate the evolution of the weather on short time scales, are just one among many processes that influence the evolution of the system, mostly by shaping distributions of water vapour and clouds. Other important processes evolve on significantly longer time scales. For instance, ocean equilibration under specified conditions (i.e. concentrations of greenhouse gases, aerosols or insolation) takes thousands of years [1]. Moreover, the climate response to changes in these boundary conditions involves processes that interact over many different time scales, so that the length of simulations useful for understanding essential questions on the climate system typically range from a few decades to a few millennia. Thus, a throughput of less than about 1 SYPD is insufficient for many applications. Workhorse climate models with a throughput of 10 SYPD are desirable, also in consideration of running large ensembles which is necessary to distinguished forced changes from natural variability [2]. The computational throughput of climate models is usually limited by their atmospheric component, which thus means that the computational requirements for numerical weather prediction and the highest-resolution general purpose climate are not that disparate.

In the Earth System, *scale interactions* are very important and scales at very different magnitude (from molecular all the way to the planetary scale) interact with each other. Numerical resolution dictates which scales and physical processes can be represented explicitly within model simulations. Processes that are not resolved explicitly need to be parametrized by adding terms to the right-hand-side of the dynamic equations, expressing the behaviour of sub-grid-scale processes on the resolved scales.

*Advances in HPC* have brought the global weather and climate prediction communities to the point where it is possible to replace various parametrizations of crucial physical processes by fluid-dynamical representations based on fundamental principles. A particular leap in this regard is the increase in global resolution and pushing the corresponding horizontal resolution to O(1 km). *Kilometre-scale global simulations* are expected to explicitly represent the transient dynamics of the largest convective clouds, gravity waves, interactions of the flow with the major orographic features [3], the evolution of fine-scale disturbances on the tropopause, important scales of surface-atmosphere interactions over both the ocean and land, as well as the scales on which weather extremes are most impactful. Simulations at this scale, with the required throughput, are becoming imaginable, given radically new algorithmic approaches and dedicated software optimization of in-production-use weather and climate models and the rise of actual exascale supercomputers. The need for these simulations is similar for weather predictions and climate projections [4] as are associated computing and data handling challenges. Moreover, ‘regional manifestations of climate change are mainly through changes in the statistics of regional weather variations’, yielding strong cross-links between weather and climate simulation and resulting—due to the required high model resolution—in an increased need for extreme HPC capacity [5]. Yet, only few scientific studies of global kilometre-scale simulations and their computational performance are available. For example, a 7 km resolution run using the NICAM model successfully simulated and reproduced life cycles of tropical cyclones, in terms of timing, motion and mesoscale structures, pointing at the prediction capabilities of global high-resolution models [6]. Concerning performance analysis, a generic performance analysis for global kilometre-scale atmospheric general circulation models was presented in [7], followed by a refined study on the matter [8], with the latter concluding that a hardware/software co-designed 28 PetaFLOP supercomputer with more than 20 million processing elements could pave the way for kilometre-scale atmospheric simulations. Subsequently, several model-specific investigations complemented the generic analysis. An idealized near-global kilometre-scale climate simulation based on the COSMO model performed at 0.043 SYPD [9] using 4888 GPU compute nodes. Further works have exploited the models NICAM (0.9 km resolution [10]), MPAS (3 km resolution, 0.16 SYPD throughput on full NERSC Edison system [11]) and SAM (4 km resolution [12]).

Yet, exascale supercomputers bring up several challenges. These include fundamental changes in node-level design, increased need for resilience methods to handle system software and hardware failures at scale, and efficient programming strategies to cope with, among others, aforementioned aspects. Concerning node-level design, due to energy limitations and further design criteria, nodes are expected to be equipped with hundreds of compute cores or accelerator architectures such as GPUs. Although it is not clear yet, how this will finally affect the design and implementation of future weather and climate models, it inevitably brings up the need to fundamentally understand the performance of weather and climate models on current architectures and to predict their performance on new architectures, as well as on ever-increasing compute resources.

The Centre of Excellence in Simulation of Weather and Climate in Europe (ESiWACE) sets up and evaluates global kilometre-scale weather and climate demonstrator simulations. These demonstrators, built on top of weather and climate models that are heavily employed in forecast production schedules and science, explore the models' computational potential and limits on current petascale supercomputers and enable insight into expected performance on upcoming exascale systems. First steps towards model intercomparison with regard to computational and scientific performance are currently being taken in the scope of the international project DYnamics of the Atmospheric general circulation Modeled On Non-hydrostatic Domains (DYAMOND, see https://www.esiwace.eu/services/dyamond for details).

*This paper* provides insights into the computational performance of two models at global near-kilometre scale: the Integrated Forecast System (IFS), and the ICOsahedral Non-hydrostatic (ICON) model; we focus on atmosphere-only simulations in the following. Both ICON and the IFS are at the heart of production schedules, with the IFS being used at the European Centre for Medium-Range Weather Forecasts and ICON at the Deutscher Wetterdienst. ICON and the IFS (the latter in the context of EC-Earth) are also used for climate projections, and both models are subject to ESiWACE investigations; ICON further participates in DYAMOND runs. We provide performance numbers (in terms of SYPD), discuss comparisons with performance models and give extrapolations towards expected performance at exascale. The discussion of model performance will be based on the target to reach at least 1 SYPD throughput in cloud-resolving simulations. This target will hardly be sufficient to fulfil the needs of *all* applications of weather and climate simulations. Long integrations over several millennia will certainly not be possible at this throughput rate. It will also not be sufficient for global ensemble simulations if a large fraction of the machine will be required to perform a single simulation. One SYPD will, however, be sufficient for *some* useful applications in operational weather or climate predictions such as deterministic global weather forecasts or climate simulations up to a length of a couple of decades. One SYPD would also allow model tuning as well as a proper quality evaluation in research experiments. The goal of 1 SYPD should therefore not be considered as the final goal for scalability but rather as a benchmark for useful model configurations.

After a review of scales relevant to weather prediction and climate projections and a detailed discussion on the great relevance of kilometre-scale global simulations (§2), challenges from computational and modelling perspectives are presented (§3). Computational performance results for the IFS and ICON high-resolution demonstrator simulations as well as first DYAMOND runs are presented and analysed in §4. We discuss performance model approaches and arising performance extrapolations for numerical weather and climate simulations at exascale in the same section. In particular, we construct simple, yet effective performance models for ICON, which on the one hand allow to predict the performance of ICON at larger node counts and, on the other hand, will be advantageous to explore ICON performance and scalability on new, exascale-relevant hardware architectures in the future. Given the importance of achieving true kilometre-scale simulation capability for a qualitative leap in weather and climate modelling, these estimates yield insights into what can be expected from these simulations at exascale on the one side and for strategic planning in Earth system model development, scientific code development and HPC system configuration on the other side. This is complemented by demonstrating the expected benefits of high-resolution simulations on science cases by a preliminary analysis of the impact of convection-resolving versus parametrized configurations.

## 2. Why the kilometre scale matters

**Figure 1.**
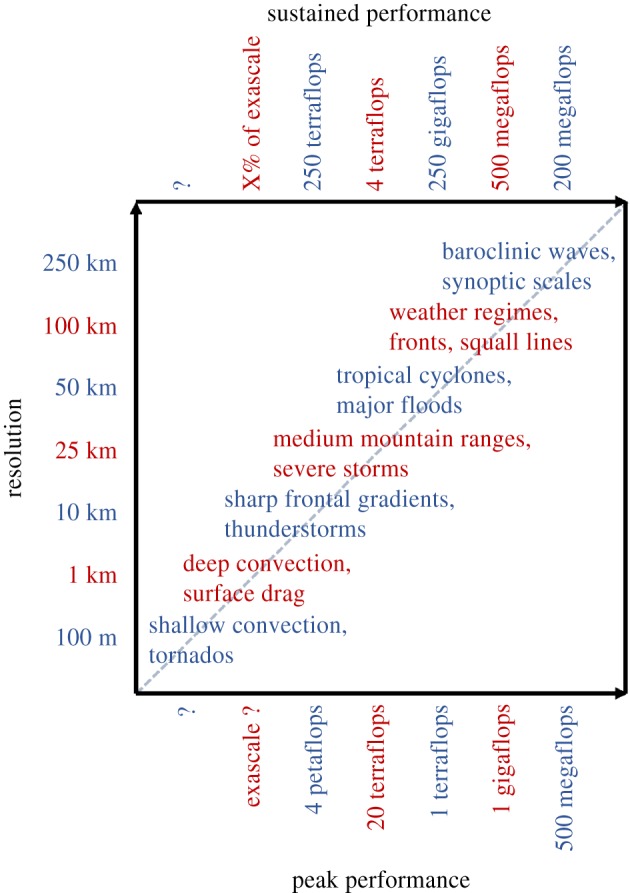
Scales in weather and climate prediction versus computational resources required to resolve them. (Online version in colour.)

The atmosphere and ocean encompass fluid-dynamical motions on scales that span more than 10 orders of magnitude, from millimetre scales where motion is randomized into thermal energy, to planetary scales where flows are influenced by the shape of the Earth itself. But not all scales matter to the same degree, as specific processes arise at specific scales; see figure 1 for an overview of 100 m–250 km scales and related processes. Modern numerical weather prediction and climate modelling, for instance, were both founded on the realization that the transient dynamics of baroclinic storms in the mid-latitude could be computed, rather than represented statistically, given a relatively coarse computational mesh [13,14]. The ability of computing machines capable of resolving processes on these scales enabled an explicit representation of the storms that are important to the weather of the mid-latitudes, as well as equator to pole energy transport which is crucial for the climate. The idea of computing weather and climate on the kilometre scales offers similar breakthroughs, as outlined below.

In the tropics, the dominant mode of energy transport is in the vertical, through deep moist convection—‘hot-towers’ [15]. These circulations, whose scale is determined by the depth of the tropical troposphere (15–20 km) transport energy from the surface to the atmosphere, where it is either then lost through radiation or exported to the extra-tropics. How this transport transpires shapes weather and major features of the climate system. By virtue of being much smaller than the depth of the troposphere, kilometre-scale simulations can represent their transient dynamics, and thereby break the deadlock that global models have since the very first days of their development. Likewise in the ocean, at the kilometre scale, ocean eddies become explicitly resolved. The effect of eddies on the climate is less researched, but may be of similar importance as is the representation of moist convection for the atmosphere, because these eddies strongly influence the stratification of the southern ocean, which then determines how much carbon and energy it takes out of the atmosphere.

Resolving major topographic or bathymetric features also means that how the mean flow interacts with the surface, and the effect of this interaction on the properties of the mean flow in the atmosphere, or on water mass formation in the ocean, becomes resolved and constrained by the laws of fluid dynamics. Being able to do away with not only convective and eddy parametrizations, but also artificial representations of ocean surface properties, or parametrizations of orographic influenced mixing and drag, thus brings simulations much closer to first principles.

No longer needing to parametrize processes important for tropical weather and global climate would certainly represent a breakthrough. Nonetheless, some might be tempted to argue that remaining parametrizations, like those of turbulent mixing processes, or cloud microphysical processes will remain, and limit the fidelity also of kilometre-scale models. What this argument fails to appreciate is that even for these parametrizations, the kilometre scale is a game changer, as it addresses the under-determination problem that limits existing parametrizations of these processes. This is the problem whereby even if a process is in principle parametrizable if the quantities that the parametrization depends on are not available, but rather the output of other parametrizations, then it compounds the difficulty of what is already a very difficult problem. As an example, trying to parametrize cloud formation processes without knowledge of the magnitude or size of the updraft in which the cloud forms is manifestly more difficult than in the case where this information is at hand, which it would be (albeit, for smaller scales, in distorted form) in kilometre-scale models. Yet, the parametrization of remaining (i.e. still unresolved) processes (such as cloud microphysics) will rise in importance, respectively, in kilometre-scale simulations.

Finally, kilometre-scale models allow an explicit representation of those processes whose impact communities are so interested in. Examples include damages from wind gusts, or hail formation or orographically influenced deluges. Such scales also begin to capture the interaction of weather and climate with urban landscapes or water ways with its sea-walls or levies.

Fundamentally, there is nothing inherently magic about models running on grid meshes of exactly 1 km. Many of the processes mentioned above begin to be represented in so far as the model mesh is considerably smaller than the process in question. This means that, e.g. a 3 km model substantially differs from a 10 km model, even at a qualitative level. Similarly, 1 km simulations show significant differences from 3 km runs. Yet, 3 km simulations resolve many of the important processes already: ironically, this also gives a particular meaning to the 1 km model, as coarser resolution versions such as the 3 km case are expected to yield new insights and will be—once the 1 km case can execute at acceptable throughput rates—ready for data assimilation and ensemble predictions.

Reaching the kilometre scale also brings the 100 m scale into view, at which it becomes possible to dispense of all parametrizations of convection, including those used to represent the large-eddies in the convective boundary layer or the shallow clouds that are a bane for those interested in Earth's climate sensitivity.

## 3. High-resolution weather and climate modelling: scalability, modelling, resolution

### (a) Scalability challenges

Since the early work of Smagorinsky, one could argue that global climate models have made incremental progress, as they have laboured to increase their resolution from 500 km to 5 km. This hundred-fold increase in scale necessitated a roughly million-fold increase in computational power (see e.g. [16] for details), a huge investment with incremental benefits as with increasing resolution not a single parametrization could be eliminated. These improvements have placed us at a threshold whereby a further jump in scales from 10 to 1 km makes it possible to dispense with parametrizations (see §2).

Part of the challenge of increasing resolution is that for explicit numerical methods, it necessitates computing smaller time steps, resolving smaller time scales and satisfying numerical stability criteria. Besides, scaling out a model at fixed resolution (often referred to as strong scaling, that is using more computational cores at a fixed number of grid points) is limited at some point: you will not get your result faster by adding further compute cores beyond that point, simply because there is not enough work per compute core left, communication between cores dominates and load imbalances between cores become more expressed.

Hence, given a (bigger, yet) fixed number of grid cells at prescribed high resolution and significantly more time steps to be calculated on each grid point, the wall clock time needed per model run increases as soon as the strong scaling limit is reached, and the result of a numerical experiment takes longer. In conclusion, if we specify the number of simulated years per wall clock day which we need to achieve to do useful science with sufficient throughput, this limits the number of grid cells that we can afford and thus the number of compute cores we can use efficiently. Of course, we can try to remedy this through performance optimization of the model per grid cell. But naturally, this approach is limited and does not mitigate the existence of a fundamental limit in resolution. This issue has recently been further elaborated in [17], motivating the application of multi-scale computing and corresponding (hybrid) simulation approaches. Owing to the strong impact of various modelling assumptions and corresponding physical processes (see §2), this seems, however, out of scope for many climatological questions. Another option to speed up high-resolution simulations on extreme scale platforms is given by parallel-in-time approaches. Despite analysis and first studies on instability problems that some parallel-in-time algorithms such as Parareal encounter for hyperbolic problems [18], an immediate use of these methods is not expected to be realizable soon for entire Earth system models.

### (b) Modelling challenges

Improving and optimizing model configurations to run as efficiently as possible on emerging supercomputers is a challenge—in particular, given the parallel use of an ever-increasing number of processing units within a single million lines-of-code simulation. The use of scalable algorithms that run efficiently on hundreds of thousands of processing units is imperative throughout the entire model configuration and global communication between processors needs to be avoided wherever possible. This is particularly difficult to realize for the development of efficient time-stepping schemes. Explicit time-stepping schemes that act locally and avoid global communication require the use of very short time steps. On the other hand, implicit or semi-implicit schemes require global communication for linear solvers but allow much longer time steps (often a factor 10 in comparison to explicit methods), see [19] for a detailed discussion.

Numerical methods that are used in weather and climate models also need to be stable and are required to work efficiently on the sphere while topography should be resolved at a resolution as high as possible on a given grid. There are several methods that compete for spatial discretization (finite differences, finite volume, finite elements, spectral element models) and different shapes of grids that are used (icosahedral, Gaussian, cubed sphere, yin yang, fully unstructured), see for example [20].

Atmosphere models are not isotropic in three dimensions. The vertical dimension is special due to stratification, lower velocities and the main direction of physical processes such as radiation and cloud dynamics. As horizontal resolution is increased, a use of higher resolution in the vertical is also required and the limit of very high resolution will require a better representation of three-dimensional effects. While physical parametrization schemes are calculated within independent vertical columns of grid cells today, more communication between columns may be required in the future; for example, if the three-dimensional shape and reflection of clouds or the angle of the sun compared to the heating on the ground needs to be taken into account in three-dimensional radiation. For simulations at very high resolution, the hydrostatic approximation, which is still used in many global weather and climate models, will break down. However, there is still discussion about the exact level of resolution at which non-hydrostatic equations are mandatory.

It is also a challenge to understand and improve the coupling between different model components (such as atmosphere, ocean, sea ice and land surface) as well as the coupling between the dynamical core and physical parametrization schemes. There is hope that an increase of resolution to kilometre-scale will improve the coupling between topography and convection, and medium and large-scale dynamics. However, the use of grids that are only able to represent deep convection explicitly may cause an incorrect energy distribution in the vertical during the transition towards cloud-resolving models. Additionally, scalability needs to be achieved for all components, in particular, also for the wave model and the ocean component—which can generate a significant ratio of the cost for weather and climate simulations—as well as the advection of tracers if a complex representation of atmospheric chemistry is used.

### (c) Remark on nominal and effective resolution

While the grid spacing is typically used to define the ‘model resolution’ (as also done in this work), the scale of the smallest processes that are fully resolved within a simulation—the effective resolution—is significantly larger. The effective resolution can be derived, for example, from comparing kinetic energy spectra between models and observations (e.g. [21,22] and references therein). The point where the modelled spectrum starts deviating from the observations indicates a lack of representation of processes at smaller scales. This is the result of a range of mechanisms such as the choice of spatial discretization and time steps, a smoothing of the orography to improve numerical stability, the effect of physical parametrizations [23] and other diffusion mechanisms. For spectral models like the IFS also the difference between the spectral resolution for the governing equations and grid-point resolution for advection and physics parametrizations needs to be considered [24]. The nominal-effective resolution difference of the IFS is about a factor of 6 and for ICON about a factor of 7 [25]. Similar factors have been derived from an assessment of mesoscale models [26].

Achieving an effective resolution of 1 km may therefore require a nominal resolution of 150 m or so. A factor of 6–7 in resolution roughly translates to a factor of 300 in computing cost counting the change in the number of grid points and time-step size. Only judging computing cost figures based on nominal resolution can therefore be misleading. To avoid merging these approximate factors with exact runtime measurements for the IFS and ICON, the following assessment is based on nominal resolution set-ups.

## 4. State of ICON and IFS global high-resolution models: model features and computational performance

### (a) ICON

ICON uses a finite difference approximation of the non-hydrostatic equations of atmospheric motion [25,27]. For global simulations, Earth is discretized via refinement of an initial globe-covering icosahedron. This refinement results in *ca* 21 million horizontal cells (triangles) to reach a global resolution of *ca* 5 km and a time step of 45 s. The number of vertical levels has been varied, with performance numbers reported in this paper using 62, 137 or 90 levels. The latter corresponds to the operational weather forecast configuration at Deutscher Wetterdienst and to the DYAMOND configuration which is being used for intercomparison with other European and international models in the near future. The sub-grid physical processes for clouds, boundary-layer mixing, radiation and the surface are described using the parametrizations of the limited-area kilometre-scale numerical weather prediction with the COSMO model [28]. The grid resolution allows to resolve convection and gravity waves, which usually are sub-grid in global weather and climate simulations. The configuration is comparable to the one described in [29] for regional simulations with the same model. In the DYAMOND set-up, data output has been enabled, providing insight into the challenges and performance limits of the high-resolution data avalanche. Three-dimensional state variables (velocities, temperature, pressure, specific humidity, cloud water, ice) are collected every hour, selected horizontal quantities (2D) every 15 min; GRIB files are written per simulated day, resulting in *ca* 164 GB output per day in the 5 km case. A detailed description of the I/O configuration, the placement of the vertical levels used in the DYAMOND run, etc. is provided online (https://www.esiwace.eu/services/dyamond-specific-pages-and-material/icon-vertical-grid). The other simulations were conducted excluding I/O. Simulations were performed on the supercomputer Mistral, partition compute2, hosted at DKRZ. Each dual-socket node of the partition features two Broadwell CPUs with 18 compute cores and a minimum of 64 GB main memory. Hyperthreading was enabled and 6 OpenMP threads per MPI process were employed.

### (b) IFS

IFS is a global, spectral model for which some of the prognostic fields are represented in a set of global basis functions (so-called spherical harmonics). The standard configuration of IFS is solving the hydrostatic equations. However, a non-hydrostatic version of IFS is also available. Typical numbers for vertical levels are 60, 91 and 137. For operational weather forecasts at ECMWF, IFS is using a TCo1279 grid (approx. 9 km horizontal resolution) with 137 vertical levels for a 10-day deterministic forecast and a TCo639 grid (approx. 18 km horizontal resolution) with 91 vertical levels for ensemble forecasts with 50 ensemble members plus one control forecast for 15 days. Atmosphere-only simulations use time steps of 450/240/180/120 s at 9/5/2.5/1.25 km resolution.

### (c) Model feature: resolving convection

**Figure 2.**
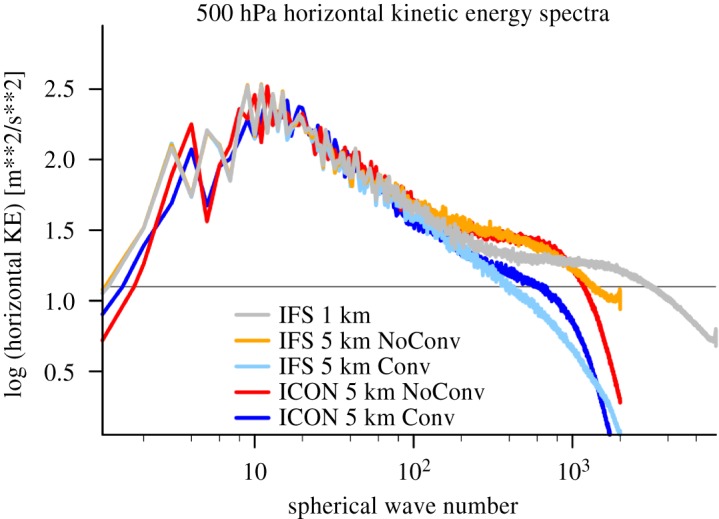
Energy spectra in 1 km and 5 km scale global IFS and ICON simulations. For the 5 km cases, simulations with and without convection parametrization are reported.

Moist deep convection and gravity waves change from being sub-grid at about 10 km grid spacing to being resolved (at least in a later development phase) at about 1 km grid spacing. The intermediate range from 1 to 10 km in resolution is often referred to as the ‘convective gray-zone’, where bulk formulae for convective parametrization schemes break down because an insufficient number of cells are present within a single grid cell, while resolution does not suffice to properly represent convection explicitly. This is demonstrated in the energy spectra of horizontal kinetic energy at 500 hPa for simulations with and without a representation of sub-grid convection for two models in figure 2. While the simulations with a convection parametrization have too little energy on scales between 20 and 200 km, the simulations without a parametrization have too much energy on the same scales relative to a 1 km simulation. This is due to the still under-resolved processes at 5 km grid spacing, which leads to an accumulation of energy on scales predetermined by the chosen grid size. While the effective resolution of the parametrized 5 km models is lower by a factor 4–8 (deviation from −5/3 spectra at lower wave numbers), the incorrect spectral energy redistribution can negatively feedback on the synoptic scales and subsequently impact on the predictability [23]. The energy spectra indicate that an even higher resolution than 5 km is necessary to properly resolve deep convection.

**Figure 3.**
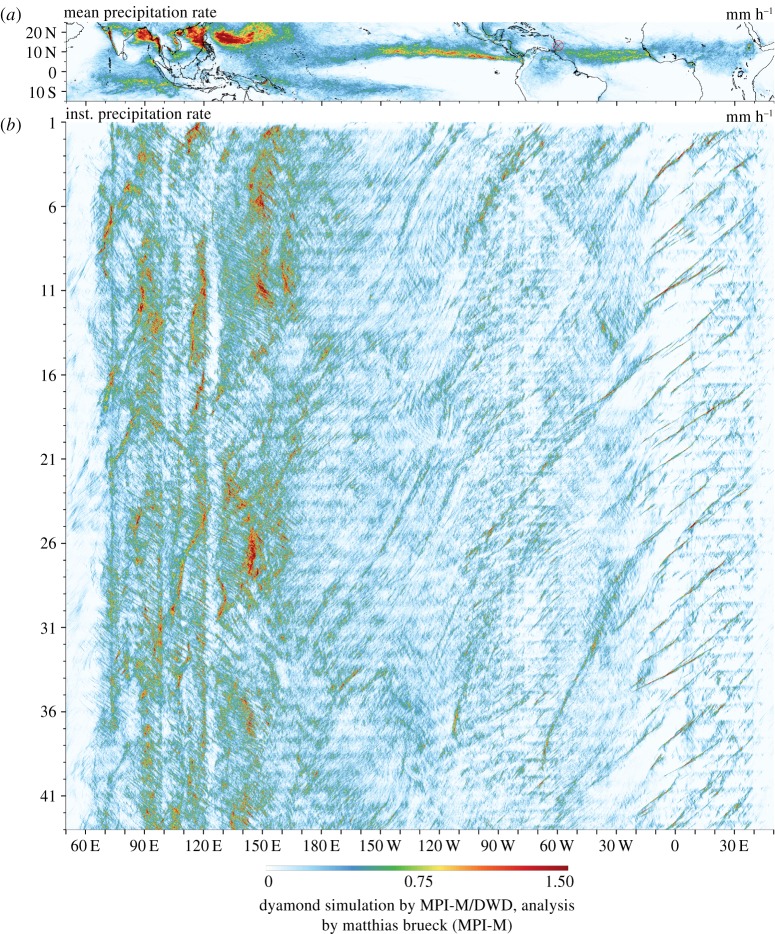
Tropical precipitation resulting from explicit convection in the ICON-DYAMOND simulations. (*a*) Temporal mean precipitation rate. (*b*) Hovmoeller diagram of meridionally averaged precipitation (longitude (*x*), time in days (*y*)). The emerging dynamic character of propagating connective clusters manifests in slated lines of precipitation.

Other aspects already improve when the sub-grid representation of convection is disabled. Interactions of the dynamic process with its environment are explicitly modelled which leads to the emergence of the full dynamic structure of convection. One example can be observed in figure 3 which shows the space–time variability of tropical convection from 15S to 25N (centring on the inter-tropical convergence zone). The precipitation rate is derived from 15 min increments of accumulated precipitation of the ICON-DYAMOND simulations. The data have first been coarsened to 0.1° and then meridionally averaged. Mesoscale convective systems, which parametrized models struggle to simulate, are visible in figure 3 as the rain bands in the time-longitude representation of precipitation. Long standing problems, like the diurnal cycle of convection over land, or parts of the large-scale structure of the general circulation improve significantly when convection is resolved.

### (d) Performance measurement and extrapolation

**Figure 4.**
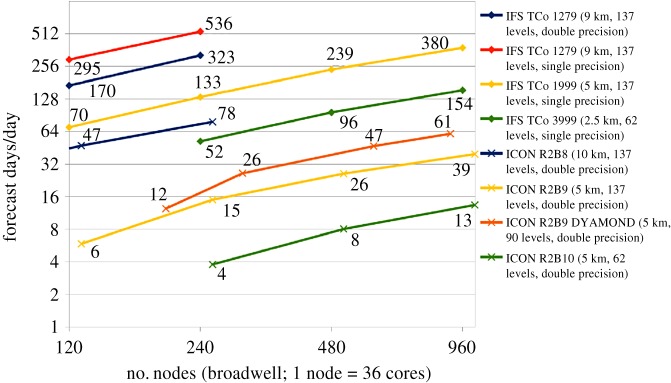
Strong scalability of IFS and ICON models in global high-resolution simulations.

Strong scalability of ICON and IFS are provided in figure 4 for global configurations ranging from 10 km to 2.5 km resolution. All performance numbers that are reported within this section relate to the throughput of the entire atmosphere-only global simulation, including dynamical core, radiation and physics components, but excluding I/O. The performance difference between the models can be explained by mainly three (partly multiplicative) factors: IFS makes use of the hydrostatic model formulation instead of the non-hydrostatic model option, which in the IFS approximately halves the performance at the moment (approx. 2×), IFS runs at single-precision (approx. 1.6×) [30] and most importantly employs a significantly longer time step due to its semi-implicit time integration (IFS/ICON: 240 s/45 s = 5× in the 5 km case). All these accumulate to a factor 16 and the figure implies about a factor 10, which is likely due to the fact that in ICON, there are different time steps used in the coupling to the physics compared to the dynamics part (the same in IFS).

Figure 4 suggests that a throughput of 1 SYPD is achievable on today's supercomputers for a 5 km resolution case (IFS, single-precision, hydrostatic, 960 nodes). Referring to the double-precision, non-hydrostatic, full I/O performance of the DYAMOND 5 km run using ICON and restricting considerations to atmosphere-only simulations, a throughput of *ca* 0.17 SYPD is reached on 900 compute nodes. A straight-forward extrapolation of the performance of the DYAMOND 5 km runs to the 1 km case corresponds to multiplicative factors in compute time of 25 in terms of horizontal resolution, 2 in vertical resolution to target for 180 vertical levels (the exact number of levels will in the end also depend on the application and adjustments of the model top) and 5 in terms of required time steps, resulting in a shortfall of 1500 to reach the target of 1SYPD (assuming sufficient memory)—of which the horizontal and vertical resolution factors 25 × 2 can potentially be directly compensated for through more computational resources in the weak scaling sense. Similar estimates for atmosphere-only simulations have recently been derived for the COSMO model and the IFS [16]. At this stage, the exact complexity of a model simulation at 1 km horizontal resolution is still not settled in terms of the complexity of model components such as the land surface, the sea ice or atmospheric chemistry or in terms of the number of model levels and the exact time-step length. The choices in this paper may therefore appear rather arbitrary. However, we have based our choices, such as 180 vertical levels, on experience from state-of-the-art models for both global and limited-area simulations [31] and are confident that the suggested complexity would result in simulations at 1 km resolution with high fidelity.

The compensation through more compute resources results in a shortfall of 30 using 25 × 2 × 900 = 45 000 compute nodes of the models, as long as I/O can be distributed correspondingly, which is far from trivial, considering the widening gap between memory/storage performance and compute power.

### (e) Performance modelling

Given the need for higher effective and, thus, nominal resolution, the scalability studies for global high-resolution cases (cf. figure 4) and the need to estimate performance on varying hardware architectures in view of the upcoming, yet unknown, exascale systems, we developed a hardware-aware performance model [32,33] for ICON and evaluated it on the supercomputer Mistral. The model takes into account data transfer in terms of cell, vertex, edge exchange between neighbouring domains, network latency and bandwidth. Owing to the explicit time-stepping scheme and the used spatial discretization, it is sufficient to restrict considerations to ICON's nearest neighbour communication. Let

$$t(N)=t_{\mathrm{comput}}(N)+t_{\mathrm{commun}}(N),$$

represent a decomposition of the total simulation time *t* into communication and computation phase, using *N* processes. Within a time step, every ICON process communicates with all of its neighbours *P* times, sending and receiving messages of size *s_p_* in communication step *p*. Assuming the simulation to be limited by the slowest process, a single send/receive command in communication step *p* can be approximated as

$$\frac{\text{ma}{\text{x}}_{n}(s_{p}(n))}{b(s_{p})}+t_{l},$$

with incremental bandwidth *b*(*s_p_*), Mistral's latency *t_l_* = 2.7*e* − 6*s*, and the maximum message size computed over all processes *n* = 1, … , *N*. Note that the bandwidth typically depends on the size of messages (the bigger the messages, the higher the bandwidth) and that the incremental bandwidth needs to be computed from the actual, measured bandwidth values by taking into consideration *t_l_* (table 1). To determine the actual bandwidth per message size, we employ a quasi-linear regression on measured bandwidth data, assuming a dependence of the form *a*_0_ · log(*s*) + *a*_1_.

**Table 1.** Measured transfer times and bandwidths on supercomputer Mistral, and derived incremental bandwidth *b*(*s*)*.*

message size *s* (MB)	transfer time (s)	bandwidth (MB s^−1^)	*b*(*s*) = *s*/((1/*B*(*s*))*s* − *t*_l_) (MB s^−1^)
0.032768	1.42 × 10^−5^	2308	2850
1.048576	2.10 × 10^−4^	4984	5049

A simple model to predict the total communication time arises as

$$t_{\mathrm{comm}}(N)=m\cdot \overset{P}{\underset{p=1}{\sum }}\text{mx}nb_{p}(N)\cdot \left(\frac{\text{ma}{\text{x}}_{n}(s_{p}(n))}{b(s_{p})}+t_{l}\right),$$

with the number of executed time steps *m* and mx*nb_p_*(*N*) denoting the maximum number of neighbours that at least one of the *N* processes features. The computation time might be expected to scale perfectly, yielding

$$t_{\mathrm{comput}}(N)=\frac{t_{\mathrm{comput}}(1)}{N}.$$

However, load imbalances per process may occur due to irregular domains and corresponding decompositions. In particular, the computational load varies significantly, for example, between cloudy and cloud-free areas. In this case, it makes sense to model

$$t_{\mathrm{comput}}(N)=\frac{t_{\mathrm{comput}}(1)-t_{\mathrm{imbalance}}(1)}{N}+t_{\mathrm{imbalance}}(N)$$

with temporal evolution *t*_imbalance_(*N*) for the imbalanced compute part. The latter is, however, very complex to deduce analytically, due to the aforementioned state-dependency argument on cloudy areas and the fact that simulation models comprise up to millions of lines-of-code.

Given a new piece of hardware and its hardware specifications, *b*(*s_p_*) and *t_l_* are fixed. Measuring performance of ICON on one node of the new hardware fixes *t*_comput_(1) and is already sufficient to evaluate scalability via the first performance model. Measuring ICON in few configurations allows to approximate *t*_imbalance_(*N*), as detailed in the following, and closes the second performance model. Thus, the models described above are simple, yet effective means to investigate performance portability—an urgent need for complex weather and climate models in the dawning of the exascale era. Since the ICON-DYAMOND 5 km configuration needs to be executed on at least 100 nodes due to memory requirements, we use the compute time on 100 nodes as a baseline instead of *t*_comput_(1) as described above. The ICON-DYAMOND configuration features 39 communication steps per time step, exchanging halo layers of cells, vertices and edges between the processes. It is further differentiated between halo1 and halo2 cell transfers, corresponding to the transfer of one or two halo cell layers. We investigated the performance of ICON-DYAMOND using 4 OpenMP threads per process. To determine *t*_imbalance_(*N*), MPI barriers and time measurements on up to 900 nodes were used. For bigger node counts, an extrapolation rule was employed.

**Figure 5.**
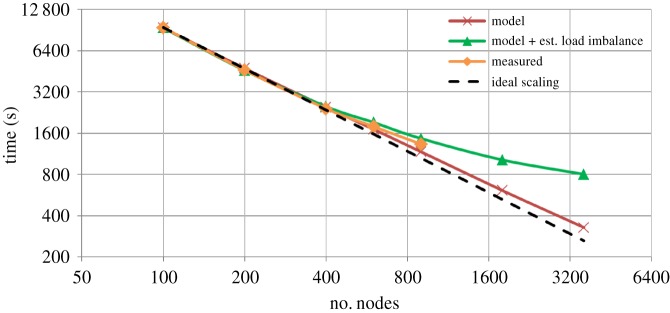
Measured (measured) and modelled performance (model, model + est. load imbalance) for the 5** **km ICON-DYAMOND configuration. Ideal scaling assumes perfect scalability of the ICON-DYAMOND 5 km simulation, taking the 100 node configuration as baseline. (Online version in colour.)

Actual execution times (measured) are compared with the two performance model predictions (model and model + est. load imbalance) in figure 5. The simplistic ‘model’ shows agreement with errors less than 13% on up to 900 nodes and predicts better scalability than featured by the actual ICON model. The ‘model + est. load imbalance’ shows slightly smaller errors (up to 10%) and predicts slightly worse scalability for ICON. Still, process-local load imbalance can be seen to be an essential component to describe a more realistic scalability behaviour on large node counts, resulting in predicted run time differences of up to 2.4× in the considered extrapolation range between the two performance models.

Considering the trends of the performance models, in particular, the ‘model + est. load imbalance’, and the measurements in figure 5, scalability tends to completely stagnate at O(4000) nodes at a performance of *ca* 0.3 SYPD. Note that this estimate corresponds to the point in the scalability curve at which a minimum time-to-solution and no more speedup is reached; typical model employments would run at lower node counts to achieve a higher parallel efficiency of, e.g. ≥ 50%. The point in figure 5 thus yields an upper bound with regard to parallelism in the model: in the present case, the arising configuration corresponds to *ca* 292 vertical columns per MPI process, or 146 columns per compute core. This is similar to previous local-area ICON simulations (*ca* 91 columns per core at a higher vertical resolution of 160 levels, using 458 752 cores see [31]). Taking up the 5-to-1 km extrapolation approach, this indicates that a 1 km ICON simulation is expected to scale up to 200 000 compute nodes—which would correspond to a pre-exascale system running at *ca* 0.22 ExaFLOP/s (extrapolated from Mistral performance, see for example, the TOP500 list entries of partitions compute1 (November 2016) and compute1 + compute2 (June 2018), www.top500.org)—and run at 0.06 SYPD (due to decreasing time-step size; assuming 180 vertical levels as sketched before).

## 5. Conclusion

Concluding, we have outlined the case for running weather and climate models on a 1 km global mesh and performed performance studies for such configurations. Our studies suggest that further performance improvements are essential to arrive at a throughput of 1 SYPD for 1 km atmosphere-only simulations at exascale. Shortfall factors have been lately reported for different models, with ICON numbers (1500×) reported in this work; for ICON, we could, based on measurements and performance models, estimate that a model performance improvement of 1SYPD/0.06SYPD ≈ 17 is necessary to arrive at the required throughput rate of 1SYPD on next-generation supercomputers, taking into account the scalability limits of the model, time step decrease with increasing resolution, etc. Achieving this in the future is further complicated through even more complex supercomputer architectures. This challenge has to be faced to understand fundamental principles in general circulation models (see §2) and, thus, to address current and future research questions, which will help to improve current forecast methodology (cf. our discussion on high-resolution model features in §4).

Many components need to be integrated to achieve the goal—new algorithms for enhanced model performance and scalability, performance optimization at all levels, increased hardware–software co-design to make sure that future architectures can be exploited by weather and climate models, etc. In this contribution, we have provided scalability studies of the IFS and the ICON model, and we have presented a simple, yet effective approach to performance modelling to quantify model shortfalls, demonstrating the model's feasibility at the ICON-DYAMOND case. The performance models are further expected to be beneficial when porting the weather and climate models to new hardware architectures: based on hardware characteristics and only a few additional performance measurements, the performance models enable scalability predictions for full model runs. Yet, improved models to describe load imbalances within the models would be highly desirable. We expect our performance models and related analysis to be helpful for other groups, who face the same scalability challenges. Depending on the weather and climate models and their communication patterns, one or the other part of the performance models may require further refinement; for example, in the case of the IFS, the term *t*_commun_(*N*) would require additional modelling to account for the transformations between spectral and grid-point space. Doing the performance modelling exercise for more weather and climate models or their sub-components that typically add complexity (such as chemistry, aerosol, carbon cycle, etc.) will be highly beneficial to develop a community plan towards global high-resolution modelling at exascale. In the case of the ICON-DYAMOND configuration, time spent in I/O amounted to up to 39% (on 900 nodes of Mistral). This will become even worse for simulations at higher resolutions. Thus, incorporating I/O in the performance models constitutes another requirement to successfully predict the performance of the weather and climate models in production mode at exascale. Future work will further focus on extending considerations to 2.5 km and 1 km runs and towards coupled atmosphere-ocean simulations.

## Data accessibility

Research data from ECMWF can be obtained on request through accessing ECMWF's meteorological data archive and retrieval system (MARS).
